# Quantification of tramadol and serotonin by cobalt nickel tungstate in real biological samples to evaluate the effect of analgesic drugs on neurotransmitters

**DOI:** 10.1038/s41598-023-37053-9

**Published:** 2023-06-23

**Authors:** Komal Zafar, Muhammad Wasim, Batool Fatima, Dilshad Hussain, Rubaida Mehmood, Muhammad Najam-ul-Haq

**Affiliations:** 1 Department of Biochemistry, Bahauddin Zakariya University, Multan, 60800 Pakistan; 2 HEJ Research Institute of Chemistry, International Center for Chemical and Biological Sciences, University of Karachi, Karachi, 75270 Pakistan; 3 MINAR Cancer Hospital, Multan, Pakistan; 4 Institute of Chemical Sciences, Bahauddin Zakariya University, Multan, 60800 Pakistan

**Keywords:** Biochemistry, Biomarkers, Chemistry

## Abstract

In this work, CoNiWO_4_ nanocomposite was used as an electrochemical sensor for the simultaneous electrochemical detection of tramadol and serotonin. The nanocomposite was synthesized using a hydrothermal method and characterized via XRD, SEM, TGA, Zeta, UV, and FTIR. The sensor was developed by depositing CoNiWO_4_-NPs onto the glassy carbon electrode surface. Tramadol and serotonin were detected by employing cyclic voltammetry (CV), differential pulse voltammetry (DPV), electrochemical impedance spectroscopy (EIS), and chronoamperometry. Analytes were detected at different pH, concentrations, and scan rates. The prepared sensor showed a 0–60 µM linear range, with a LOD of 0.71 µM and 4.29 µM and LOQ of 14.3 µM and 2.3 µM for serotonin and tramadol, respectively. Finally, the modified electrode (CoNiWO_4_–GCE) was applied to determine tramadol and serotonin in biological samples.

## Introduction

Opioids are analgesics used to relieve moderate to severe pains^1^. The effects of opioid overdosing are not easily detected and differentiated due to their similar effects with nervous poisoning agents^2^. Tramadol [(1R,2R)-2-(dimethylamino)methyl-1-(3-methoxyphenyl)cyclohexanol]^3^ is a prodrug acting as a weak opioid used in post-operative care to treat acute pain^1^. It also acts at µ-opioid receptor^3^ by the reuptake inhibition of noradrenaline and serotonin (5-HT)^4^ and is a synthetic analog to codeine. It is used separately or combined with non-steroidal anti-inflammatory drugs (NSAIDs) for severe pain and neurological disorders^5^. Its excessive use is prohibited due to several health issues associated with its higher concentration^6^. Tramadol overdosing may cause health issues related to the nervous system, respiratory pathways, vomiting, fatigue, nausea, and depression^7^.

Neurotransmitters are chemical messengers performing physiological and physical functions, and their functionalities are linked to CNS^8^. Serotonin, also known as 5-hydroxytryptamine (5-HT)^9^, is a monoamine neurotransmitter involved in various biological processes. It regulates sleep, enhances mood, and improves heart functioning and appetite^10^. 5-HT is a biomarker for depression and irritable bowel syndrome^11^. Human body has 10 mg 5-HT, and 2% is present in the CNS. Any change in its levels is linked to neurological disorders^12^. Normal serotonin levels in urine and serum samples range from 300–1650 nM and 270–1490 nM, respectively^13,14^, and less than 0.0568 nM in CSF^15^. Low serotonin levels in the human body may cause mental health disorders^16^, while high levels cause sudden infant death syndrome (SIDS)^17^. Serotonin syndrome, generally caused by selective serotonin reuptake inhibitors (SSRIs), is also associated with tramadol. Since tramadol is known as a serotonin reuptake inhibitor, it releases serotonin in large amounts. Using SSRIs along with tramadol produces synergetic serotonin effect^18^.

Colorimetry^19^, chemiluminescence (CL)^20^, HPLC^21–23^, spectrophotometry^24,25^, and electrophoresis^26,27^ have been employed to detect SER and TRA. However, these methods lack sensitivity and stability and are time-consuming. Therefore, there is a need to develop reliable and sensitive methods. Electrochemical detection is widely employed to monitor drug levels and biological molecules due to their easier use and less time consumption. Several reports have been published on separate electrochemical detection of TRA^5,28–30^ and SER^9,31–33^. However, no work exists on the simultaneous electrochemical detection of TRA and SER.

Recently, nanomaterials-modified glassy carbon electrodes (GCE) have been used to study the redox behavior of analytes on electrode surfaces^34^. Nanomaterials of diverse compositions have been used as potential electrode materials, including transition metal oxides, binary metallic oxides, etc. Transition metal oxides (TMOs), such as MgO_2_, NiO, and Co_3_O_4,_ are utilized due to their low cost and rich redox chemistry. Transition metal tungstate (MWO_4_), with wolframite-type geometry, earth abundance, and multiple valence states, has extensively been reported in different fields, such as water splitting, photodegradation, and sensors. The enhanced electron transport rate during electrochemical reactions depends on adding tungstate to transition metal oxides^35^. Metal tungstates are preferred due to their high charge storage capacity^36^. Cobalt has cyclic stability with good rate capability and low specific capacity, while nickel provides good specific capacitance with less rate capability^37^. Hydroxides, oxides, phosphides, and sulfides of cobalt have been used in electrochemical sensing due to their electronic properties, lower cost, and remarkable electrocatalytic activity. Literature suggests that cobalt and nickel have electrocatalytic capabilities, i.e., high surface area, elevated electron conductivity, physicochemical stability, and tunable architecture^38,39^. Both metals are eco-friendly, cost-effective, and abundant. A nickel and cobalt composite with tungsten could be useful electrode material in electrochemical sensing^36^.

Simultaneous detection of multiple analytes is a hot topic in sensing. Nada et al. fabricated and utilized cobalt oxide/ionic liquid crystal/carbon nanotubes decorated carbon paste electrode (CPE) to simultaneously detect two narcotic analgesics (tramadol and nalbuphine) in human urine samples. The proposed sensor showed good detection limits with charge transfer enhancement and remarkable conductivity^40^. In another study, tramadol and nalbuphine were simultaneously detected by Pt–Pd-/NiO-NPs/SWCNTs incorporated on CPE in the presence of binder, i.e., 1-ethyl-3-methylimidazolium methanesulfonate (EMICH_3_SO_3_^-^). The nanocomposite showed good electrocatalytic activity in real samples^41^.

Herein, cobalt–nickel bimetallic tungstate (CoNiWO_4_) nanocomposite was synthesized by hydrothermal method and employed as electrode material for the simultaneous detection of serotonin and tramadol. The nanocomposite was characterized by ultraviolet (UV) spectroscopy, X-ray diffraction (XRD), dispersive energy X-ray (EDX) spectroscopy, thermogravimetric analysis (TGA), Fourier transform infrared spectroscopy (FTIR), scanning electron microscopy (SEM) and zeta analysis. CV, DPV, and EIS were used to examine the electrochemical behavior of CoNiWO_4_. The developed sensor can simultaneously detect both analytes in a wide linear range. CoNiWO_4_ nanocomposites can detect tramadol in patients to relieve acute pain. According to our literature survey, tramadol has been detected previously; however, its effect on serotonin has never been evaluated. Therefore, we also studied the relationship between tramadol and serotonin. This relationship and simultaneous electrochemical detection have not been reported previously. Sensitivity, stability, and biological sample analysis further showed the practicality of the prepared sensor.

## Experimental

### Chemicals and reagents

Sodium tungstate dihydrate (Na_2_WO_4_⋅2H_2_O, 97.0%), cobalt chloride hexahydrate (CoCl_2_⋅6H_2_O, 99%), and nickel chloride hexahydrate (NiCl_2_⋅6H_2_O, 98%) were purchased from Sigma Aldrich. Tramadol hydrochloride (99.9%) and serotonin hydrochloride (99.9%) were obtained from Sigma Aldrich. Deionized water was obtained from the Milli-Q water purification system (Merck, Millipore). For the synthesis of 0.1 M phosphate buffer saline (PBS), the ingredients include dipotassium phosphate (K_2_HPO_4,_ 98%) and monopotassium phosphate (KH_2_PO4, 98.5%).

### Synthesis of CoNiWO_4_ nanocomposite

Cobalt nickel tungstate (CoNiWO_4_) nanosheets were prepared by adding 0.065 g Na_2_WO_4_⋅2H_2_O, 0.02 g CoCl_2_⋅6H_2_O, and 0.02 g NiCl_2_⋅6H_2_O in 100 mL distilled water under ultrasonication. The clear solution was transferred to a 150 mL autoclave, sealed tightly, and kept in the furnace for 10 h at 200 °C. Teflon was cooled to room temperature. The product was collected, washed, and dried in an oven for 6 h at 90 °C^35^.

### Characterization techniques

Nanocomposite was characterized using XRD (Bruker D8 Advance powder diffractometer) to observe the crystal structure. SEM (Scanning electron microscope JSM-7200 F JEOL Japan) and EDX (INCA X Sight Oxford Instruments) analyzed the size and morphology of synthesized material. TGA (TGA/DSC 3+ Mettler Toledo Hong Kong) checked the thermal stability. UV (AQ7100APAC Thermofisher Scientific UK), FTIR (Invenio-FTIR Spectrometer Bruker, Germany), and zeta potential (Malvern zeta-analyzer) were employed to get further information on the prepared material.

### Electrochemical detection of analytes by CoNiWO_4_–GCE

The redox behavior of CoNiWO_4_/GCE was analyzed by cyclic voltammetry on a potentiostat (PG-STAT) using three electrodes system. Pt-wire was used as a counter electrode, Ag/AgCl as a reference electrode, and modified GCE as the working electrode. GCE was cleaned with alumina slurry, sonicated in a water–ethanol mixture, and washed with water. Nanoparticle slurry was prepared, deposited on GCE, and dried in the air. Different parameters, such as concentration and pH, were optimized at room temperature. DPV was employed to investigate the simultaneous detection of tramadol and serotonin. Tramadol and serotonin solutions were prepared at different concentrations, i.e., 10 µM, 20 µM, 30 µM, 40 µM, 50 µM and 60 µM and varying pH, i.e., 6.8, 7.0, 7.2, 7.4, 7.6 and 7.8. All measurements were carried out at a scan rate of 100 mVs^−1^ at room temperature. The initial and final frequencies were kept at 100,000 Hz and 0.01 Hz, respectively. The step and modulation potentials were 0.005 V and 0.025 V, respectively. Chronoamperometry was carried out at 0.01 V for 12 h.

### Ethical consent

Samples were collected in sample tubes with the participants' informed consent after approval from the Ethical Committee of Sahiwal Medical College, Sahiwal, Pakistan. All the procedures and experiments performed in this study were according to the guidelines of the Ethical Committee.

### Serum sample collection

Blood samples of individuals with post-operative care were collected in sample tubes. Samples underwent routine hematological tests after being collected on MEL-6318J/K Hematology Analyzer (Manual Code No. 0614-004583F, International Div., Sales Promotion Section, Nihon Kohden Crop., Tokyo, Japan). The blood samples were centrifuged to obtain serum for detecting tramadol and serotonin.

## Results and discussion

### Characterization of CoNiWO_4_ nanocomposite

**Figure 1:**
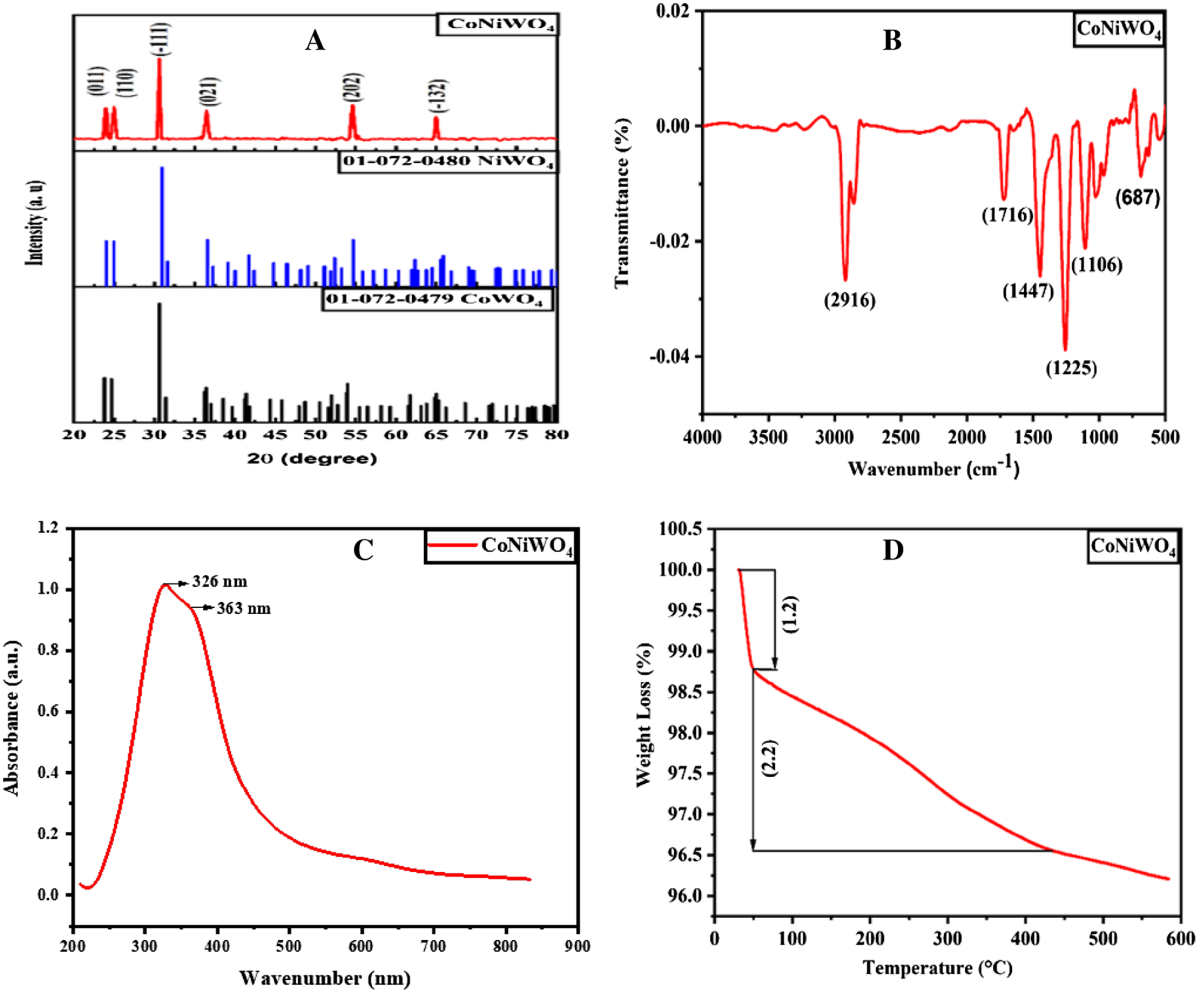
(**A**) FTIR, (**B**) XRD, (**C**) UV spectroscopy, and (**D**) TGA of CoNiWO_4_ nanocomposite.

X-ray diffraction (XRD) analysis was carried out on Bruker D8 Advance powder diffractometer to examine the phase purity and crystal structure of CoNiWO_4_, as shown in Fig. 1A. XRD pattern of CoNiWO_4_ is in agreement with standard crystal patterns of monoclinic 01-072-0479 CoWO_4_ and 01-072-0480 NiWO_4_, confirming the formation of monoclinic CoNiWO_4_. CoNiWO_4_ shows peaks at 23.9°, 25°, 30.6°, 36.4°, 54.6°, and 65°. XRD peaks are strong and sharp, indicating the good crystallinity of CoNiWO_4_. The average crystallite size was determined using the Scherrer formula as given in Eq. (1):

1 $$D=K\lambda /\beta \cdot cos\theta$$

where $\lambda$ is the X-ray wavelength, K is the Scherrer constant, $\theta$ the diffraction angle, and $\beta$ the full width at half maximum (FWHM) of the diffraction peak. The crystallite size (D) is calculated as 23.2 nm.

The inter-atomic spacing of the lattice is calculated using Bragg’s equation (Eq. 2),

2 $$n\lambda =2dsin\theta$$

Inter atomic spacing (d) is found as 3.71 Å.

FTIR spectrum of CoNiWO_4_ was recorded in the range of 4000–500 cm^−1^ (Fig. 1B). The IR bands are compared with the reference data bank, and the C=O band is observed at 1716 cm^−1^^1^. The peak intensity indicates the lower dipole moment, mainly resulting from a molecule's increasing and decreasing bond angle^2^. The bands at 2916 cm^−1^, 1716 cm^−1^, 1447 cm^−1^, 1225 cm^−1^, 687 cm^−1^ indicate C–H stretching, C=O (carboxylic acid), =CH_2_ bend, C–O in alcohols, and W–O in tungstate (WO_4_), respectively. =CH_2_ may be due to some impurity element during analysis.

**Figure 2:**
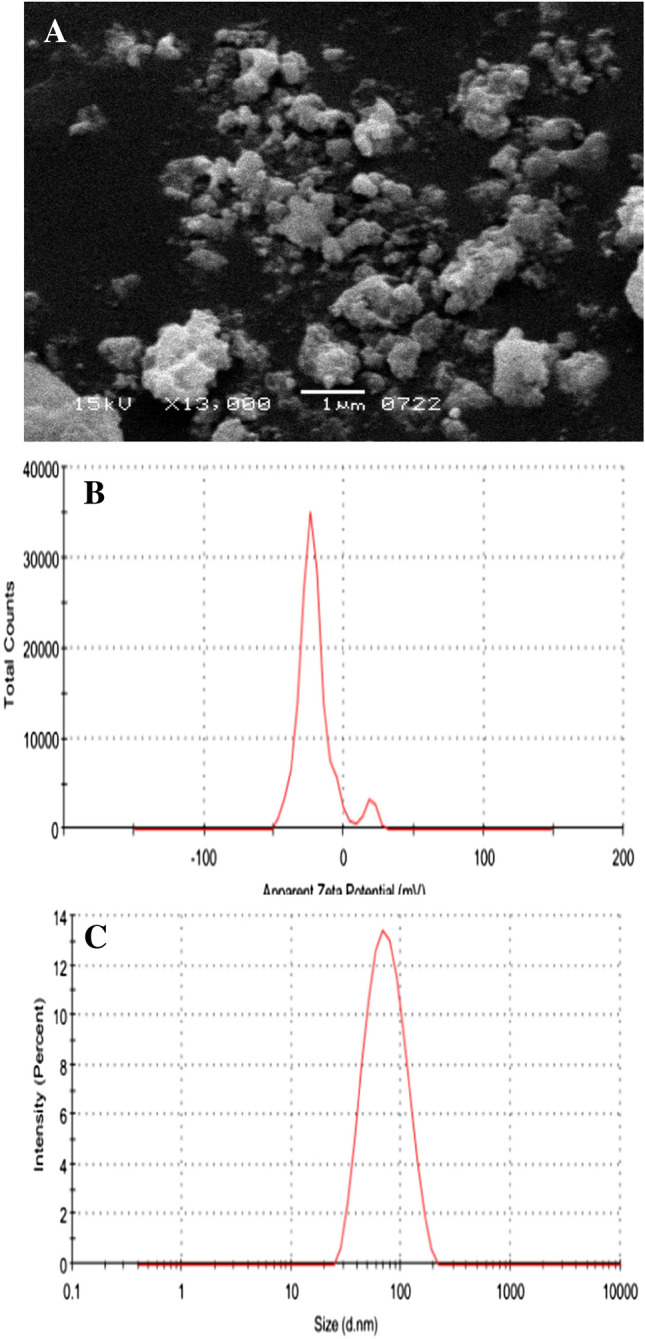
(**A**) SEM image of CoNiWO_4_, (**B**) zeta potential distribution, and (**C**) size distribution by intensity.

The UV spectrum of CoNiWO_4_ is shown in Fig. 1C. Two strong absorption peaks are observed for CoNiWO_4_ nanocomposite; one prominent peak at 328 nm and a shoulder peak at 360 nm. These absorption peaks are linked to typical forbidden d-d electronic transitions, depicting localized Co^2+^ ions^42^. The maximum absorbance at 312 nm shows the presence of metals in the nanocomposite. Cobalt and nickel fall in the UV range of 180 to 450 nm^43^, confirming their presence in CoNiWO_4_. Thermogravimetric analysis (TGA) indicates the thermal stability of CoNiWO_4_. There is gradual weight loss from ~ 50 to 450 °C, indicating that CoNiWO_4_ can withstand high temperatures (Fig. 1D). Figure 2A shows the SEM image and particle size distribution in the 90–100 nm range. The zeta potential of CoNiWO_4_ was recorded on the Malvern zeta-analyzer, as shown in Fig. 2B. The results show zeta potential of − 22.6 mV and 18.8 mV with standard deviations (SD) of 9.36 mv and 4.63 mv for two peaks and conductivity of 0.0206 mS/cm in water. The peak at − 20.5 mv indicates that negatively charged ions capped CoNiWO4 and good dispersion stability. The average zeta size is 66.50 (d nm) with a polydispersity index (PDI) of 0.173 and SD equal to 31.66 (d nm) (Fig. 2C). This PDI value indicates the system showing poly-dispersed behavior in water.

EDX results show that Co, Ni, W, and O are distributed uniformly in the sample (Fig. S1).

### Electro-oxidation mechanism of tramadol and serotonin

**Figure 3:**
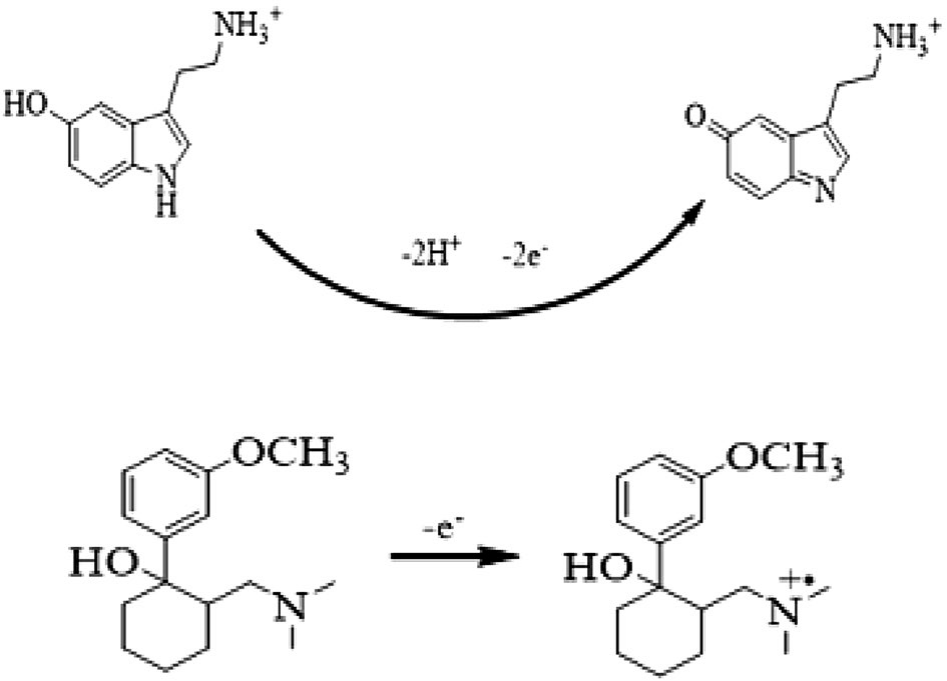
Electro-oxidation mechanisms of ser (upper equation) and tra (lower equation).

The electro-oxidation process determines the electroanalysis and sensing mechanism of analytes. The electro-oxidation mechanisms of serotonin^44^ and tramadol^3^ are given in Fig. 3.

### Electrochemical studies on CoNiWO_4_-GCE

**Figure 4:**
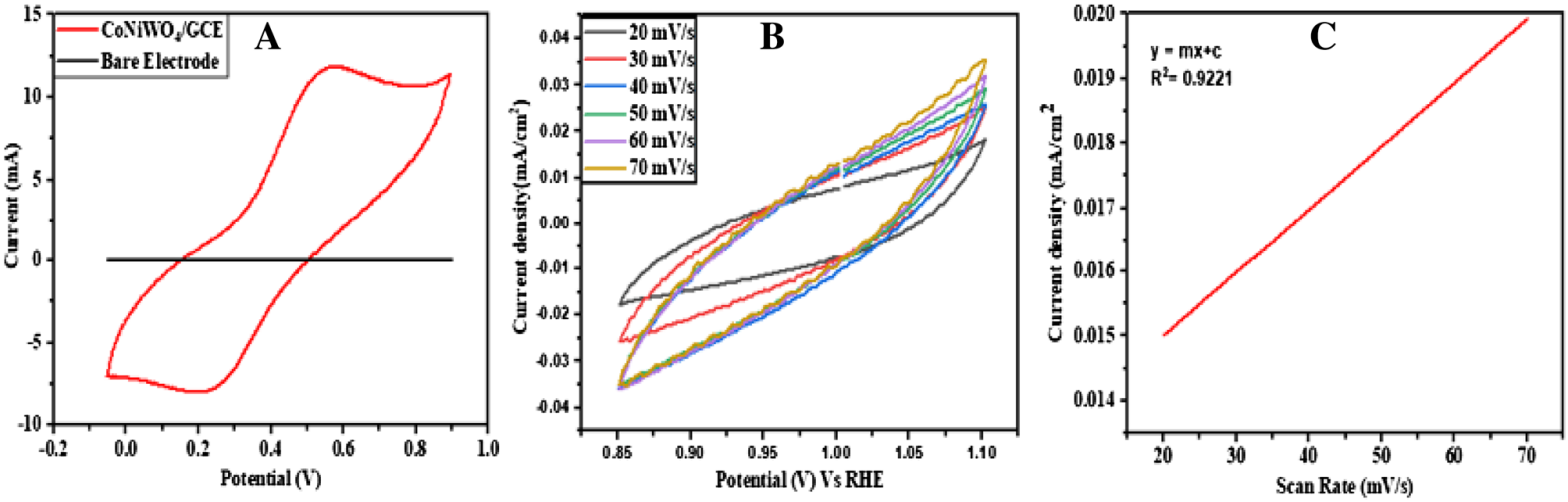
(**A**) Cyclic voltammogram showing the conductivity of CoNiWO_4_-GCE and bare electrode, (**B**) Cyclic voltammogram showing the electrochemical active surface area (ECSA) of CoNiWO_4_-GCE at various scan rates, and (**C**) The corresponding line graph.

Cyclic voltammetry and differential pulse voltammetry were employed for the electrochemical studies on CoNiWO_4_-GCE. CV analyzed the electrical conductivity of the modified electrode in 0.1 M potassium ferrocyanide solution containing KCl (0.1 M), and the electrical conductivity of the bare electrode was checked for comparison. A cyclic voltammogram (Fig. 4A) with prominent oxidation–reduction peaks of CoNiWO_4_-GCE depicts higher conductivity, while bare GCE shows no obvious redox signals, suggesting the better conductivity of the modified electrode.

### Electrochemical active surface area (ECSA) of CoNiWO_4_-GCE

ECSA of CoNiWO_4_-GCE is determined using solutions of KCl (0.1 M) and K_4_Fe(CN)_6_ (0.04 M). To determine ECSA, a linear graph is plotted between the current density and scan rate. The obtained values are then used to calculate the ECSA from the following formula. ECSA results are obtained at 20, 30, 40, 50, 60, and 70 mV/s scan rates. The slope of the curve is obtained, and ECSA is calculated as 0.671 cm^2^ for CoNiWO_4_-GCE, which is greater than that of bare GCE, i.e., 0.073 cm^2^^45^. ECSA curve for CoNiWO_4_-GCE with its corresponding line graph is given in Fig. 4B,C. Randles–Sevcik equation^46^ is applied to calculate the peak current value (Ip) (Eq. 3).

3 $$\mathrm{ECSA}=\frac{\mathrm{Cdl}}{\mathrm{Cs}}$$

$${\text{ECSA}} = 0.049/0.073$$

$${\text{ECSA}} = 0.671{\text{ cm}}^{2}$$

4 $${\text{Ip}}\upalpha = \pm \, \left( {{2}.{69} \times {1}0^{{5}} } \right){\text{ n}}^{{{3}/{2}}} {\text{AD}}^{{{1}/{2}}} {\text{Cv}}^{{{1}/{2}}}$$

$${\text{Ip}}\upalpha = 0.006 \; {\text{mA}}$$

where A is the ECSA of the modified electrode, n is the number of electrons, D is the constant, and C is the concentration of the electrolyte solution^46^.

### Electrochemical sensing of tramadol and serotonin

#### Concentration optimization

**Figure 5:**
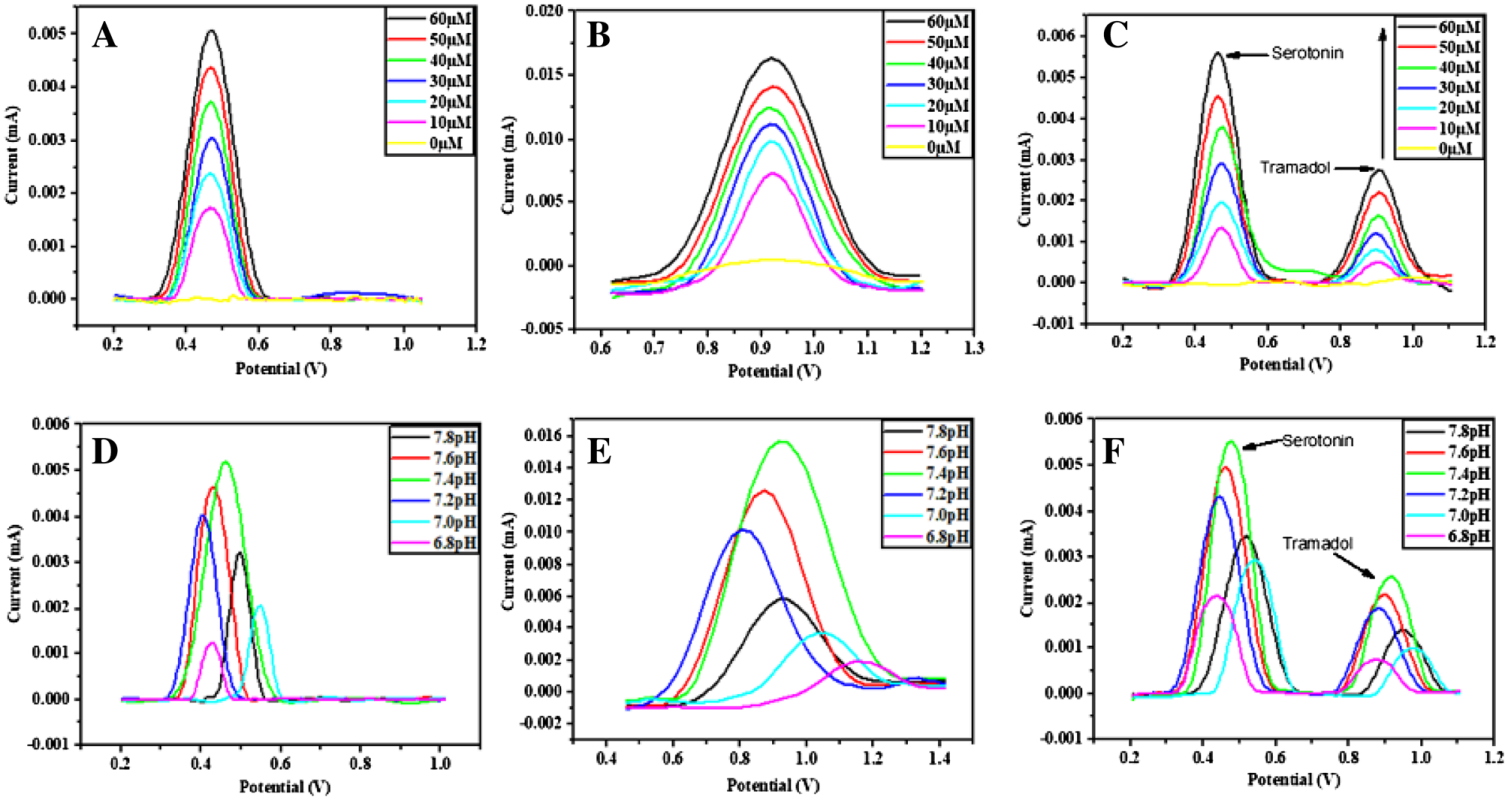
Peaks show the effects of concentrations (**A**–**C**) and pH (**D**–**F**) on the electrochemical response of CoNiWO_4_-GCE for the electrochemical sensing of Ser and Tra in 0.1 M PBS. (**A**) Effect of concentrations of Ser, (**B**) effect of concentrations of Tra, (**C**) simultaneous effect of concentrations of Ser and Tra, (**D**) effect of pH on Ser detection, (**E**) effect of pH on Tra detection, and (**F**) effect of pH on simultaneous detection of Ser and Tra.

DPV was employed for the electrochemical detection of tramadol and serotonin on CoNiWO_4_-GCE (Fig. 5). First, the analytes are detected separately, and the oxidation current peaks increased with the increase in the concentration in 0.1 M potassium phosphate buffer (pH 7.4). Sharp and intense peaks for both the analytes are observed at 60 µM showing maximum current value, while the lowest oxidation current peak is observed at 10 µM (Fig. 5A,B). A calibration plot is constructed between analyte concentration and current. The obtained linearity R^2^ is 0.9954 and 0.98789 for serotonin and tramadol, respectively, as depicted in Fig. S2A,B.

The possibility of simultaneous detection of SER and TRA is confirmed through DPV curves, keeping the concentration of one analyte constant and varying on the other. The R^2^ for serotonin, evaluated from the simultaneous detection line graph, is 0.99661, while tramadol is 0.97696. Fig S2C,D shows their corresponding line graphs. These results show that both analytes are simultaneously detected, and neither interferes with the other’s detection. A cyclic voltammogram for the simultaneous detection of serotonin and tramadol (60 µM) by employing bare GCE and CoNiWO_4_ modified GCE in 0.1 M PBS (pH 7.4) is shown in Fig. S3A. The bare electrode depicted little to no redox behavior, whereas the modified electrode indicated prominent oxidation peaks. In addition, the behavior of the modified electrode towards serotonin and tramadol via cyclic voltammetry is also represented in Fig. S3B.

#### pH optimization

pH of the solution also affects serotonin and tramadol detection. pH effect is checked using different pH buffers. Most intense peaks are observed at pH 7.4, the physiological pH (Fig. 5D–F), suggesting that physiological conditions suit the electrochemical determination of serotonin and tramadol. Although all these tests are performed under the physiological pH range, a wider pH range analysis is also performed and presented in Fig S4 to elaborate on the effect of acidic and basic conditions. Results reveal that peak position shifts considerably at very low pH due to changes in redox behavior under these conditions.

### Roughness factor (Rf)

The roughness factor, assessed by the electrochemical method, depends on the electrode size and the number of redox points on the electrode surface. Rf value is calculated as the ratio of the surface area of the modified electrode (A_2_) to the surface area of bare GCE (A_1_)^47^.

5 $$Rf=A_{2}/A_{1}$$

The Rf value was calculated as 9.1.

### Chronoamperometric analysis

Chronoamperometry evaluates the stability and activity of the designed sensor, performed for 12 h at the scan rate of 50 mV/s. The linear response shows a sudden decrease in current till 2 h and then becomes constant and stable, depicting consistency in the electrode system. The chronoamperogram is shown in Fig. S5. Similarly, cyclic voltammetry evaluated the stability (Fig. S6). The stability of modified electrode CoNiWO_4_-GCE is determined by running 100 cycles in a 60 µM solution of tramadol and serotonin in 0.1 M PBS of pH 7.4. Results indicate that CoNiWO_4_-GCE can reproducibly be used many times.

### Electrochemical impedance studies on CoNiWO_4_-GCE

**Figure 6:**
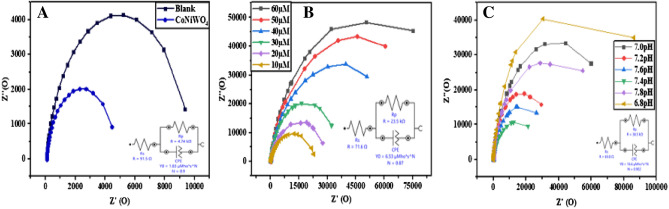
Electrochemical impedance studies, (**A**) bare electrode and CoNiWO_4_-GCE, (**B**) at various analytes concentrations, and (**C**) at different pH ranges.

Electrochemical impedance spectroscopy (EIS) is employed to determine the charge transfer process on an electrode in 0.1 M potassium ferrocyanide solution (Fig. 6). The bare electrode impedance is also compared. Bare GCE shows a large semi-circle with charge transfer resistance (R_ct_) of 9.74 KΩ, implying greater impedance. R_ct_ for the modified electrode is 4.74 KΩ indicating the greater electron transfer between electrode and analyte solution due to the higher conductivity of the modified electrode compared to bare GCE. The impedance for standard solutions of tramadol and serotonin is also determined. A big semi-circle is obtained at higher concentrations of analytes (60 µM), showing greater impedance which decreases with the decrease in analytes concentration. Impedance is minimum at the lowest concentration of 10 µM, attributed to the electrostatic interactions affecting the electron-transfer resistance of analyte recognition on the electrode surface and is directly related to the analyte concentration. Another factor that affects the rate of the charge transfer process is the pH of the analyte solution. Different pH solutions (from 6.8 to 7.8 pH range) are analyzed, and the results show minimum impedance at 7.4. This shows that pH 7.4, the physiological pH, is best suited for the maximum electron transfer rate.

### Heterogeneous electron transfer constant (K°)

The electron transfer constant is calculated using the EIS studies. In EIS, two segments are obtained, i.e., linear and semi-circle. The semi-circle measures the kinetics of electron transfer of the redox probe and is represented by charge transfer resistance (Rct). The linear segment represents diffusion at lower frequencies. The heterogeneous electron transfer constant is calculated from the given Eq. (5)^48^:

6 $$k^\circ =RT/F2RctAC$$

where R is the general gas constant, T is 298.15 K, C is the concentration of potassium ferrocyanide solution, and A is the electrochemical surface area. Rct is calculated from EIS analysis. The electron transfer constant for both bare and modified electrodes is determined as $k^\circ =$ 5.3136 × 10^–9^ cms^-1^ and $k^\circ = 6.63$× 10^–9^ cm s^−1^, respectively. The electrode system with a greater K° value achieves equilibrium in less time, indicating a fast electron transfer rate.

#### Limit of detection (LOD) and limit of quantification (LOQ)

The limit of detection is the lowest amount of analyte measured by an analytical measurement. Each analyte has its specific LOD value. LOD is measured by the following equation:

7 $$LOD \, = \, 3\;{\text{s/m}}$$

where *s* is the standard deviation of the calibration plot response obtained by constructing a linear graph of the concentration of tramadol and serotonin against generated current, and *m* is the slope. In the case of simultaneous detection, the obtained LODs of serotonin and tramadol are 0.71 µM and 4.29 µM, respectively.

The limit of quantification is the smallest amount of analyte quantified. It is measured by the following equation:

8 $$LOQ = 10\;{\text{s/m}}$$

The obtained LOQs of tramadol and serotonin in case of simultaneous detection are 14.3 µM and 2.3 µM, respectively.

#### Recovery analysis

Recovery analysis of Tra and Ser is done to examine the applicability of CoNiWO_4_-GCE for biological samples. Serum samples are diluted 20 times with PBS of pH 7.4. The recovery of analytes is determined by spiking different concentrations of standard tramadol and serotonin. The obtained recoveries range from 76.6–98.1% and 83.8–91.0% for tramadol and serotonin, respectively.

**Table:** 

Samples	Added conc (µM)	Found conc (µM)	Recovery (%)
Tramadol			
S1	60	46.9	76.6
S2	60	55.5	92.5
S3	60	58.9	98.1
Serotonin			
S1	60	54.6	91.0
S2	60	52.7	87.8
S3	60	50.3	83.8

#### Serotonin and tramadol detection in serum samples of post-operative individuals

**Figure 7:**
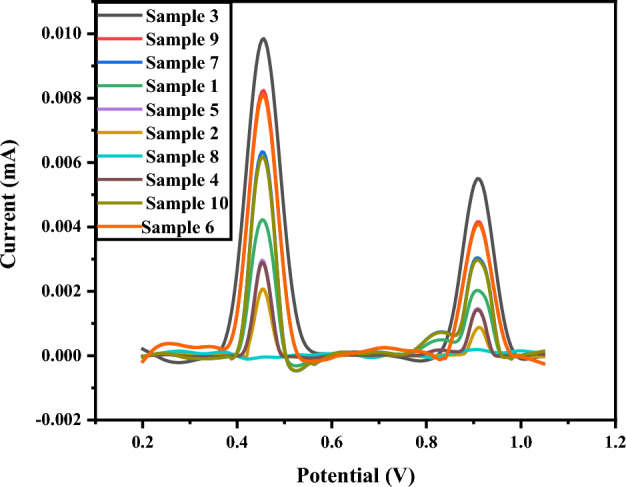
Differential pulse voltammetric results of Tra and Ser in post-operative patients obtained on CoNiWO_4_-GCE.

Tramadol is given as post-operative care to the patients to relieve pain. Tramadol increases the serotonin concentration in serotonergic neurons, and the increased serotonin levels in the body produce effects similar to pain-relieving medications. The analytes’ levels are detected in serum samples by CoNiWO_4_-GCE using DPV. Sample 1 is of a normal healthy individual, and no redox behavior is observed due to the blood's absence or lower amount of analyte. The intense peak for sample 7 indicates the higher current value due to the higher concentration of tramadol and serotonin in serum samples compared to other patient samples (Fig. 7).

It is accessed at different times to check the reproducibility and potential of the fabricated sensor for tramadol and serotonin determination in post-operative patients. The serum of patients who are administered tramadol is analyzed. As depicted in Fig. S7 (supporting information), three patients show a sharp peak during the initial hours, illustrating that tramadol concentration is the highest and reduces with time. A little variation in current is observed, as metabolic rates vary from individual to individual. This shows that fabricated sensors can be commercialized due to their reproducibility. Reproducibility studies are performed to check the potential of CoNiWO_4_ for commercialization. The modified electrode detects tramadol and serotonin in five standard solutions under the same conditions. Figure S8 represents that the fabricated sensor in standard tramadol and serotonin solution shows similar results.

## Conclusion

In this work, the electrochemical detection of two analytes, i.e., serotonin and tramadol, are reported individually and simultaneously using CoNiWO_4_ as electrode material. CoNiWO_4_ nanocomposite is synthesized using a hydrothermal method and characterized by FTIR, UV, SEM, XRD, zeta, and TGA to confirm the size, morphology, composition, and thermal stability. CV is utilized to measure the analytical parameters, while DPV is employed for the electrochemical sensing of the analytes at different concentrations and pH ranges. The stability of the modified electrode is checked by chronoamperometry. Finally, analytes are checked in the serum samples of post-operative patients, and results reveal that the amounts of both tramadol and serotonin in these patients are higher than in normal healthy individuals. CoNiWO_4_ provides easy, safe, reliable, cost-effective, electrochemically stable, and selective material for the simultaneous electrochemical detection of tramadol and serotonin.

## Supplementary Information

Supplementary Figures.

## Data Availability

All data generated or analyzed during this study are included in this article.
