# Supplementary material for: Quantification of tramadol and serotonin by cobalt nickel tungstate in real biological samples to evaluate the effect of analgesic drugs on neurotransmitters
Source: Sci Rep. 2023 Jun 23;13:10239. doi: 10.1038/s41598-023-37053-9 (PMC10290146; doi:10.1038/s41598-023-37053-9)
Supplement: Supplementary file 1 — Supplementary Figures. [file 41598_2023_37053_MOESM1_ESM.docx]

***Supporting Information***

**Effect of Analgesic Drugs on Neurotransmitters: Quantification of Tramadol and Serotonin by Cobalt Nickel Tungstate in Real Biological Samples**


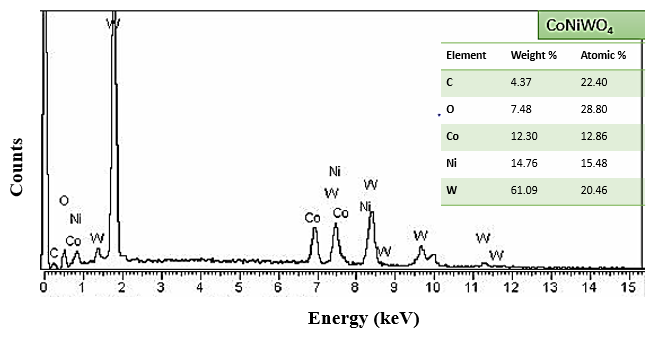


***Fig. S1.*** *EDX results showing the elemental composition of CoNiWO_4_.*


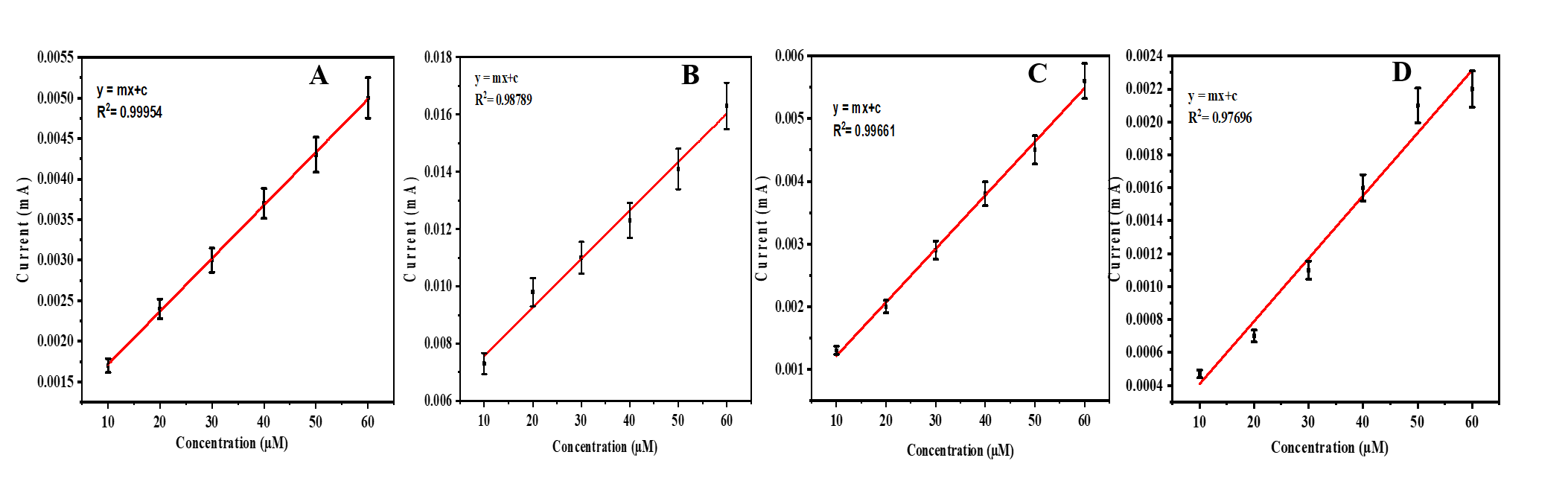


**
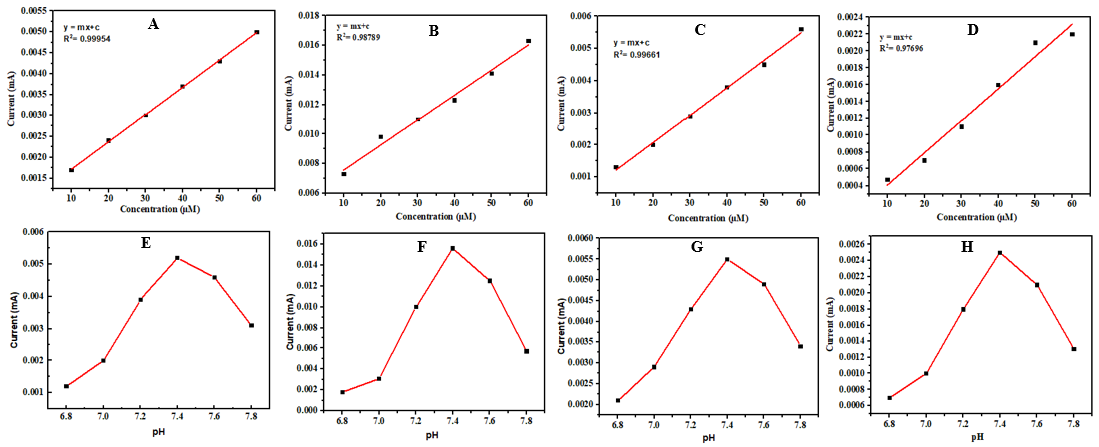
**

***Fig. S2.*** *Calibration curves for the concentrations and pH of serotonin and tramadol in individual and simultaneous detections, (A-D) shows calibration curves for concentration while (E-F) shows calibration curves for pH.*

**A**


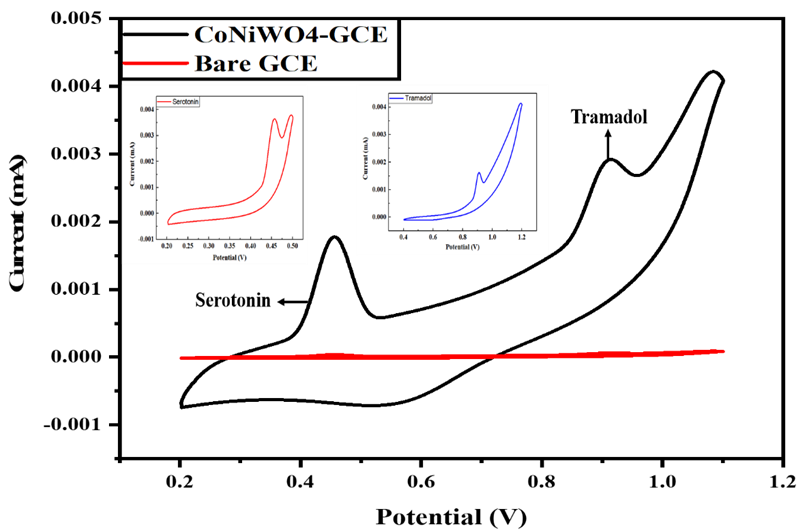


**B**

**Fig. S3.** Comparative study of the behavior of the analytes in bare and modified electrodes (A) DPV and (B) CV analysis.

**Fig. S4.** Effect of wider pH range on electrochemical detection of serotinin and tramadol.


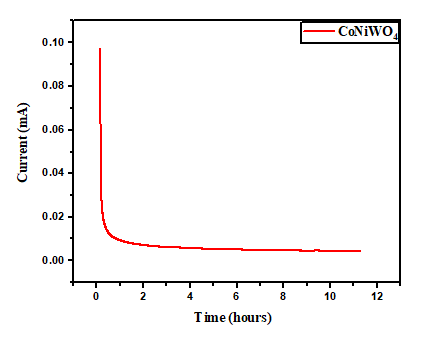


***Fig. S5.*** *Chronoamperometry showing the stability of CoNiWO_4_-GCE*

***Fig. S6.*** *Cyclic voltammetry analysis of serotonin and tramadol for the stability of CoNiWO_4_-GCE.*


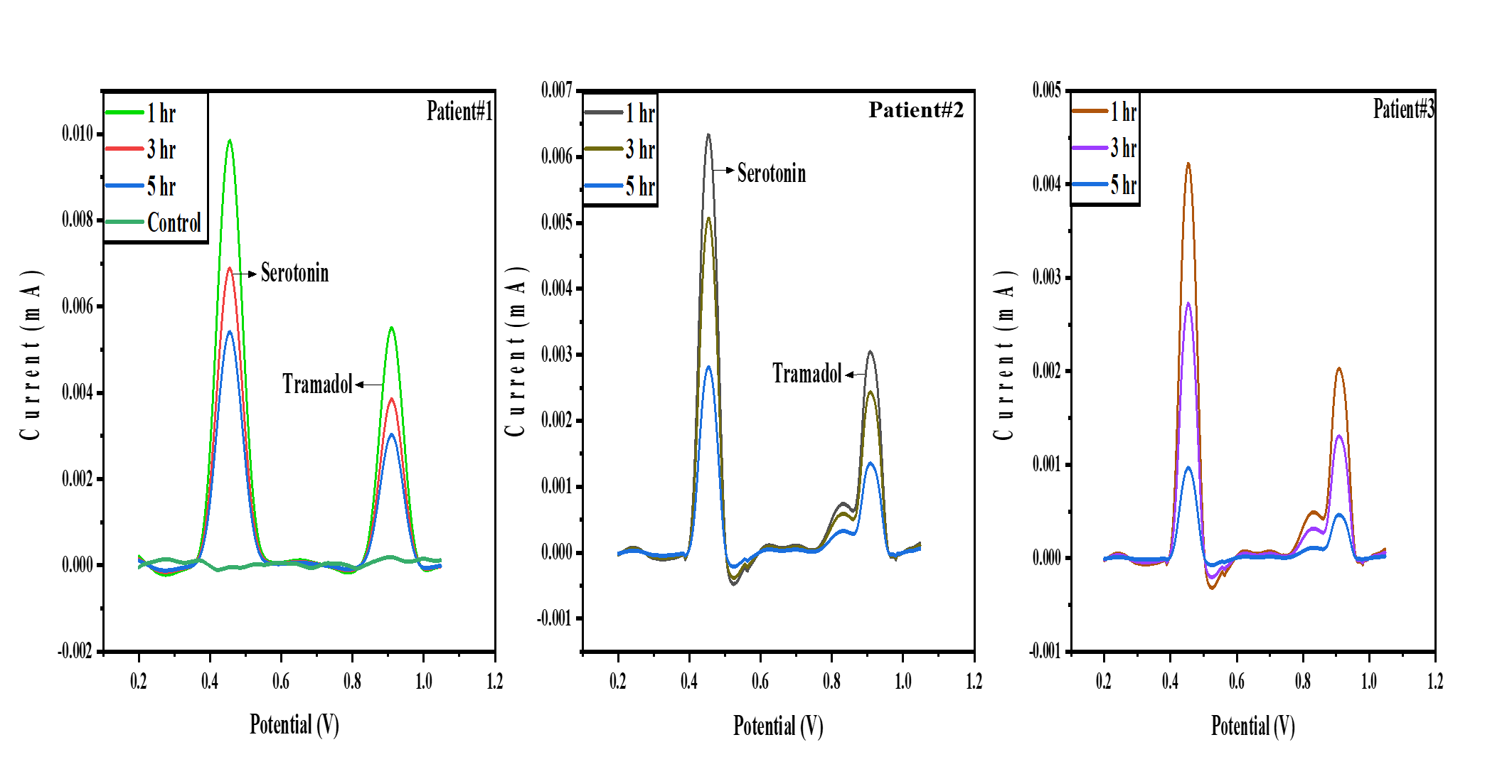


***Fig S7.*** *Reproducibility of the modified electrode for serotonin and tramadol analysis in serum samples.*

**Fig S8.** Reproducibility studies on CoNiWO_4_ modified electrode for tramadol and serotinin detection under optimized conditions.
